# Supplementary figures and images for: An unexpected role for the conserved ADAM-family metalloprotease ADM-2 in Caenorhabditis elegans molting
Source: PLoS Genet. 2022 May 31;18(5):e1010249. doi: 10.1371/journal.pgen.1010249 (PMC9187072; doi:10.1371/journal.pgen.1010249)

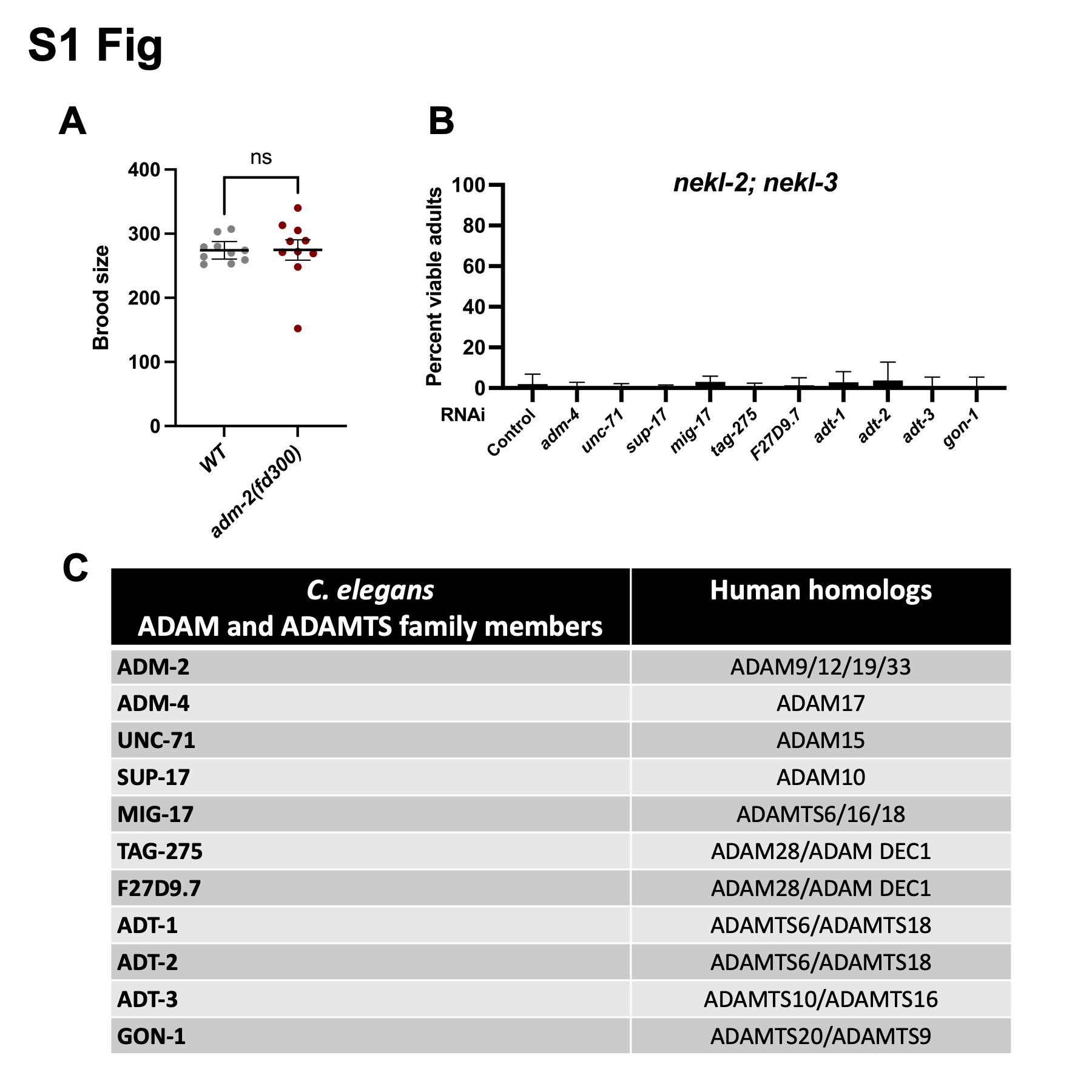

Supplement: S1 Fig — (A) Dot plot showing average brood sizes for 10 individual wild-type and adm-2(fd300) mutant worms. (B) Bar plot showing the failure of most C. elegans ADAM family members to suppress molting defects in nekl-2; nekl-3 mutants. (C) Table showing ADM-2 C. elegans orthologs and their corresponding human homologs. Error bars in A, B represent 95% confidence intervals. p-Values were determined using an unpaired t-test (A) (ns, p ≥ 0.05) or Fisher’s exact test (B): ****p ≤ 0.0001. Raw data for this figure is provided in S1 File. (TIFF) [file pgen.1010249.s001.tiff]

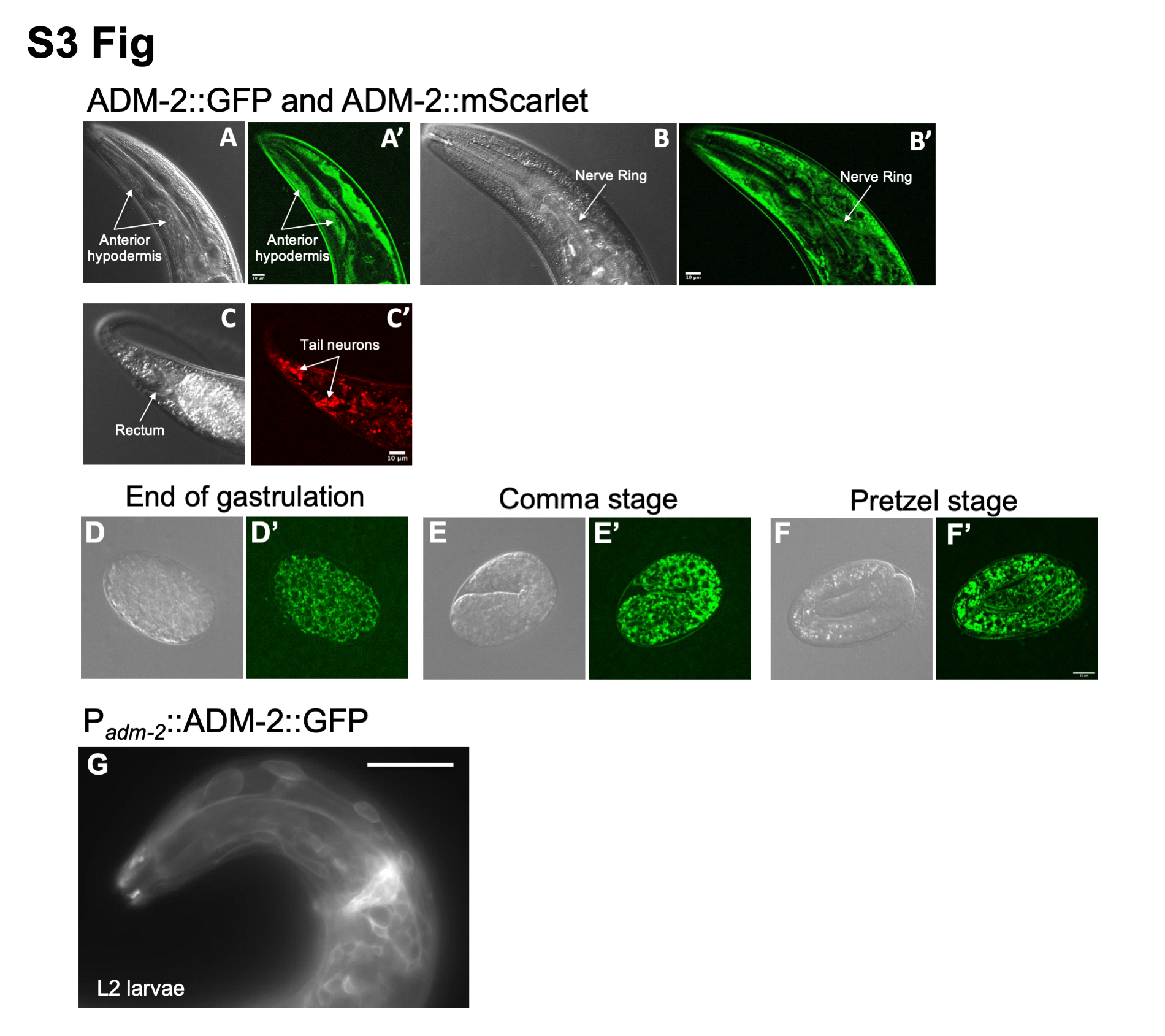

Supplement: S3 Fig — (A–F and A’–F’) Representative DIC (A–F) and confocal (A’–F’) images of ADM-2 expression showing the anterior hypodermis (A, A’), nerve ring (B, B’), tail neurons (C, C’), and various stages of embryonic development (D–F’). Bar in A’ = 10 μm (for A, A’); in B’ = 10 μm (for B, B’); in C’ = 10 μm (for C, C’); in F’ = 10 μm (for D–F’). (G) Representative confocal image of an L2 larva expressing multi-copy Padm-2::ADM-2::GFP in the plasma membrane of head neurons. Bar in G = 25 μm. (TIFF) [file pgen.1010249.s003.tiff]

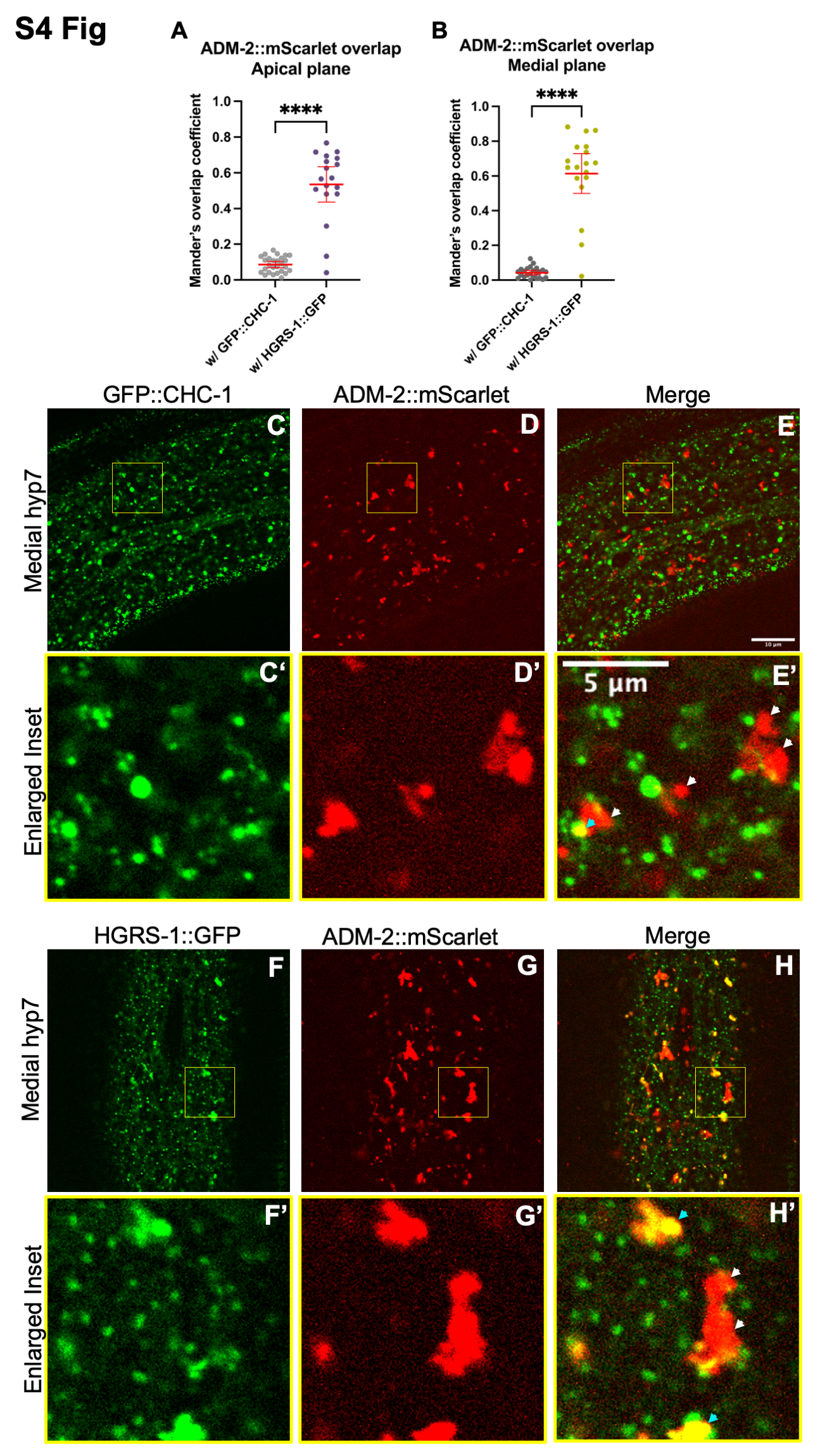

Supplement: S4 Fig — (A, B) Dot plots showing quantification of Mander’s overlap coefficient for the overlap of ADM-2::mScarlet with GFP::CHC-1 and Phyp7::HGRS-1::GFP proteins within the apical (A) and medial (B) planes. Mean values and 95% confidence intervals (error bars) are indicated. p-Values were calculated using an unpaired test: ****p ≤ 0.0001. (C–H) Representative confocal images of GFP::CHC-1 (C), Phyp7::HGRS-1::GFP (F), and ADM-2::mScarlet (D, G) within the hyp7 medial plane. C’–H’ are insets of C–H confocal images. Bar in E = 10 μm (for C–H); in E’ = 5 μm (for insets C’–H’). (E’,H’) White arrows show ADM-2 large vesicular structures that do not colocalize with GFP::CHC-1 and Phyp7HGRS-1::GFP puncta, which are lysosomes. Cyan arrows indicate vesicles containing ADM-2 that colocalize with GFP::CHC-1 and Phyp7::HGRS-1::GFP. Raw data for this figure is provided in S1 File. (TIFF) [file pgen.1010249.s004.tiff]

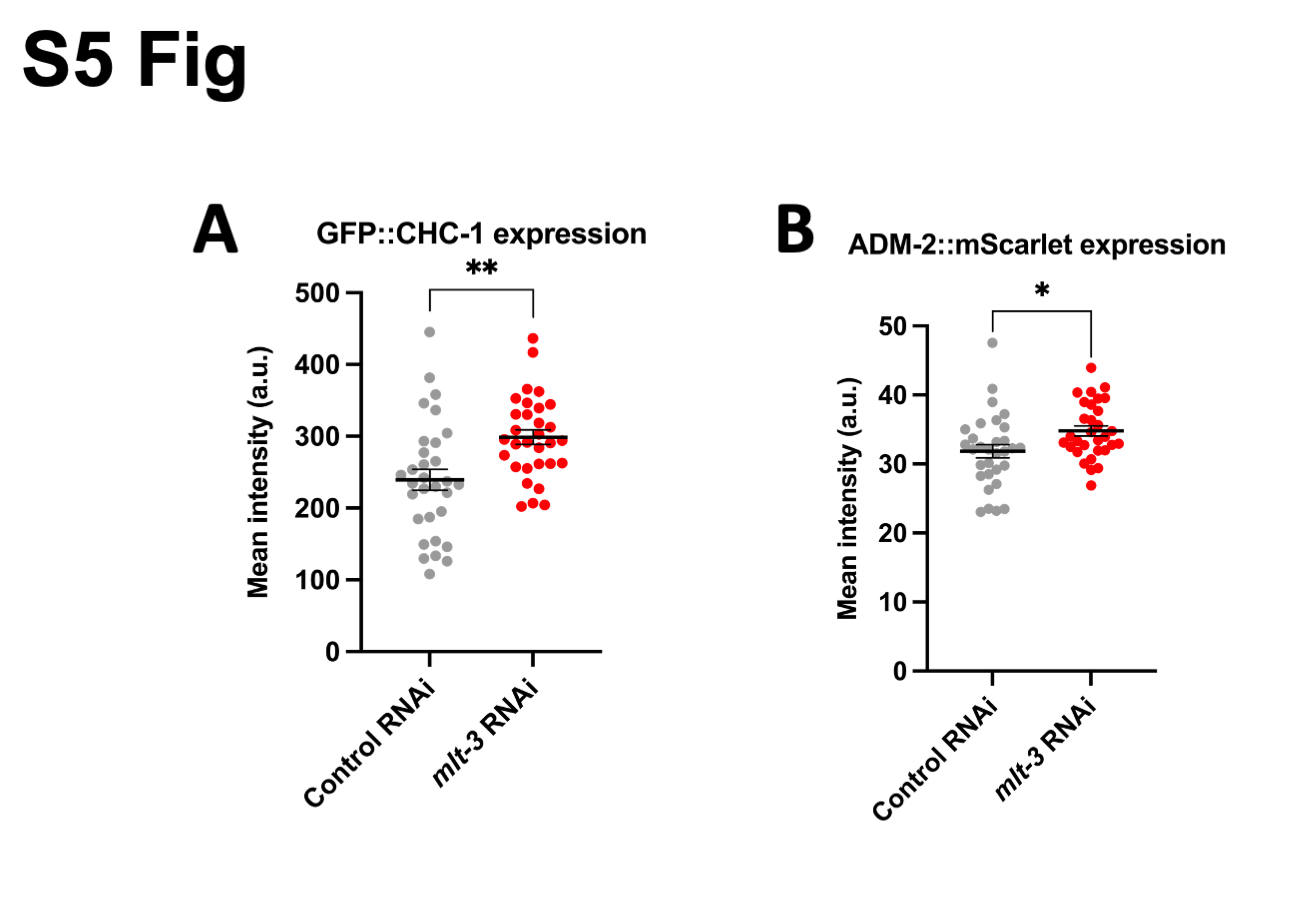

Supplement: S5 Fig — (A,B) Dot plot showing the mean intensity (a.u.) of GFP::CHC-1 (A) and ADM-2::mScarlet (B) expression in the presence of Control RNAi (i.e., empty vector) and mlt-3 RNAi. Group means along with 95% confidence intervals (error bars) are indicated. p-Values were obtained by comparing means using an unpaired t-test: **p ≤ 0.01, *p ≤ 0.05. Adult worms were imaged for this experiment. Raw data for this figure is provided in S1 File. (TIFF) [file pgen.1010249.s005.tiff]

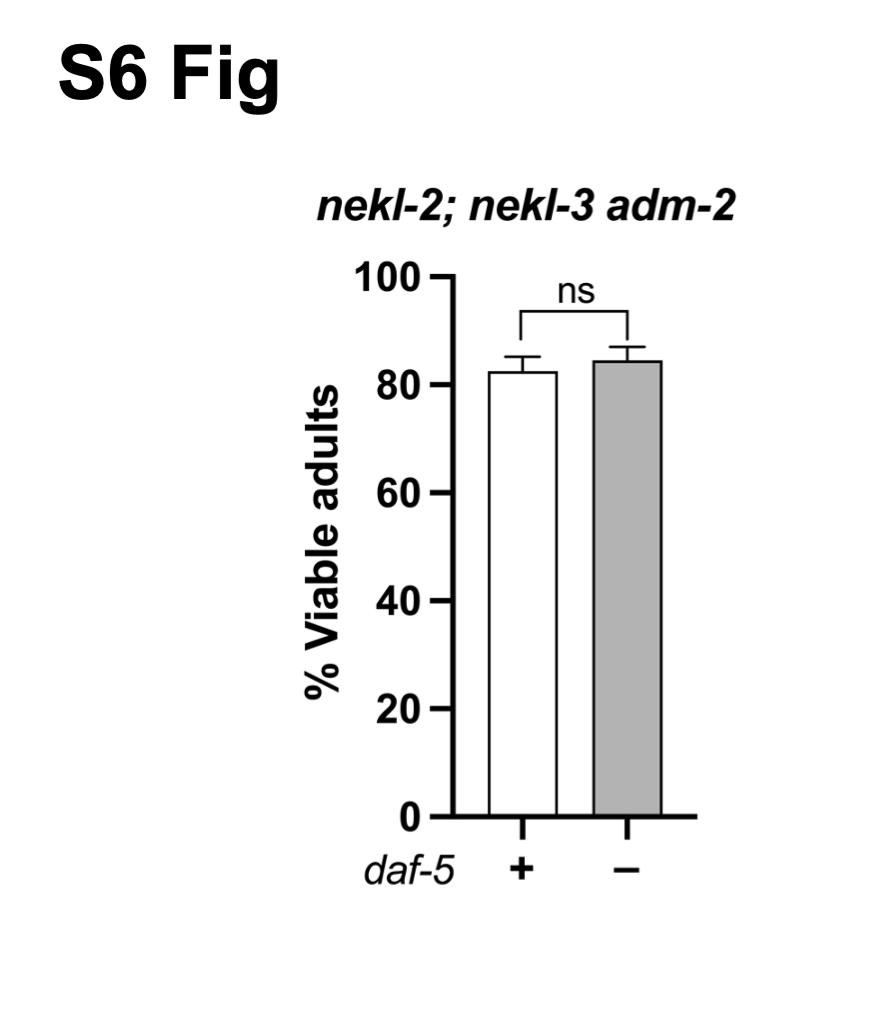

Supplement: S6 Fig — Bar plot showing suppression in nekl-2; nekl-3 adm-2(fd130) in the presence and absence of daf-5(e1386). Loss of DAF-5 leads to strong defects in the induction of the dauer pathway. p-Values were obtained by comparing means using Fisher’s exact test. ns, p > 0.05. Raw data for this figure is provided in S1 File. (TIFF) [file pgen.1010249.s006.tiff]

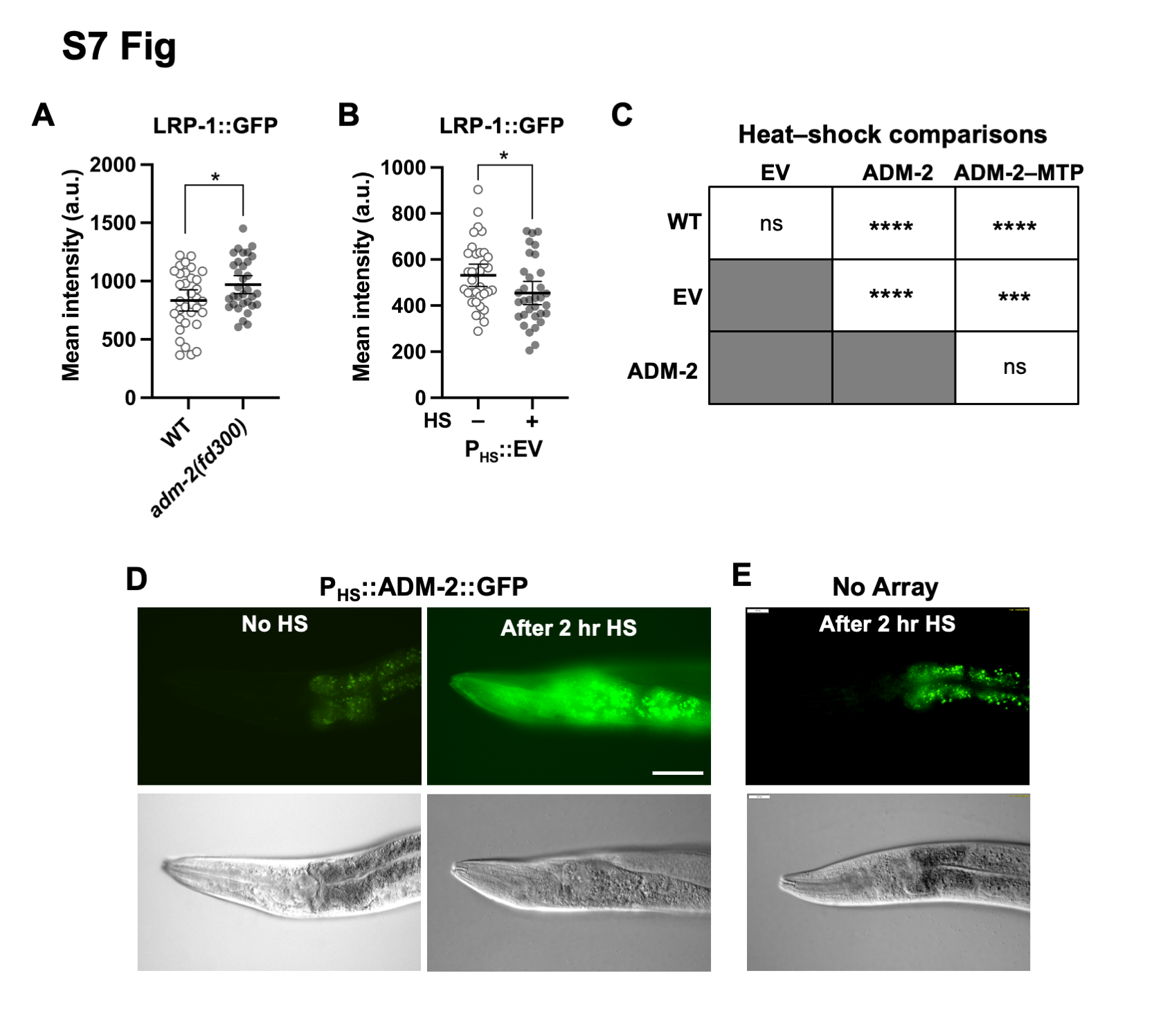

Supplement: S7 Fig — (A) Dot plot showing LRP-1::GFP mean intensity (a.u.) within the apical plane for each individual L4 worms in wild type and adm-2(fd300) backgrounds. (B) Dot plot showing LRP-1::GFP mean intensity (a.u.) within the apical plane for adults containing the Phsp-16::Empty vector transgene in the absence of heat shock and after heat shock. Group means along with 95% confidence intervals (error bars) are indicated in plots A and B. p-Values were obtained by comparing means using an unpaired t-test: *p ≤ 0.05. (C) p-values obtained from comparisons of LRP-1::GFP mean intensity (a.u.) values in strains with the indicated transgenes after heat shock. ****p ≤ 0.0001; ***p ≤ 0.001, ns, not significant. (D) Florescence and corresponding DIC images of an adult worm carrying Phsp-16::adm-2::gfp in the absence of heat shock and after heat shock. (E) Florescence and corresponding DIC images of an adult worm without the heat-shock array after heat shock. Scale bar in D = 50 μm (For all images in D). Scale bar in E = 20 μm (For images in E). Raw data for this figure is provided in S1 File. (TIFF) [file pgen.1010249.s007.tiff]

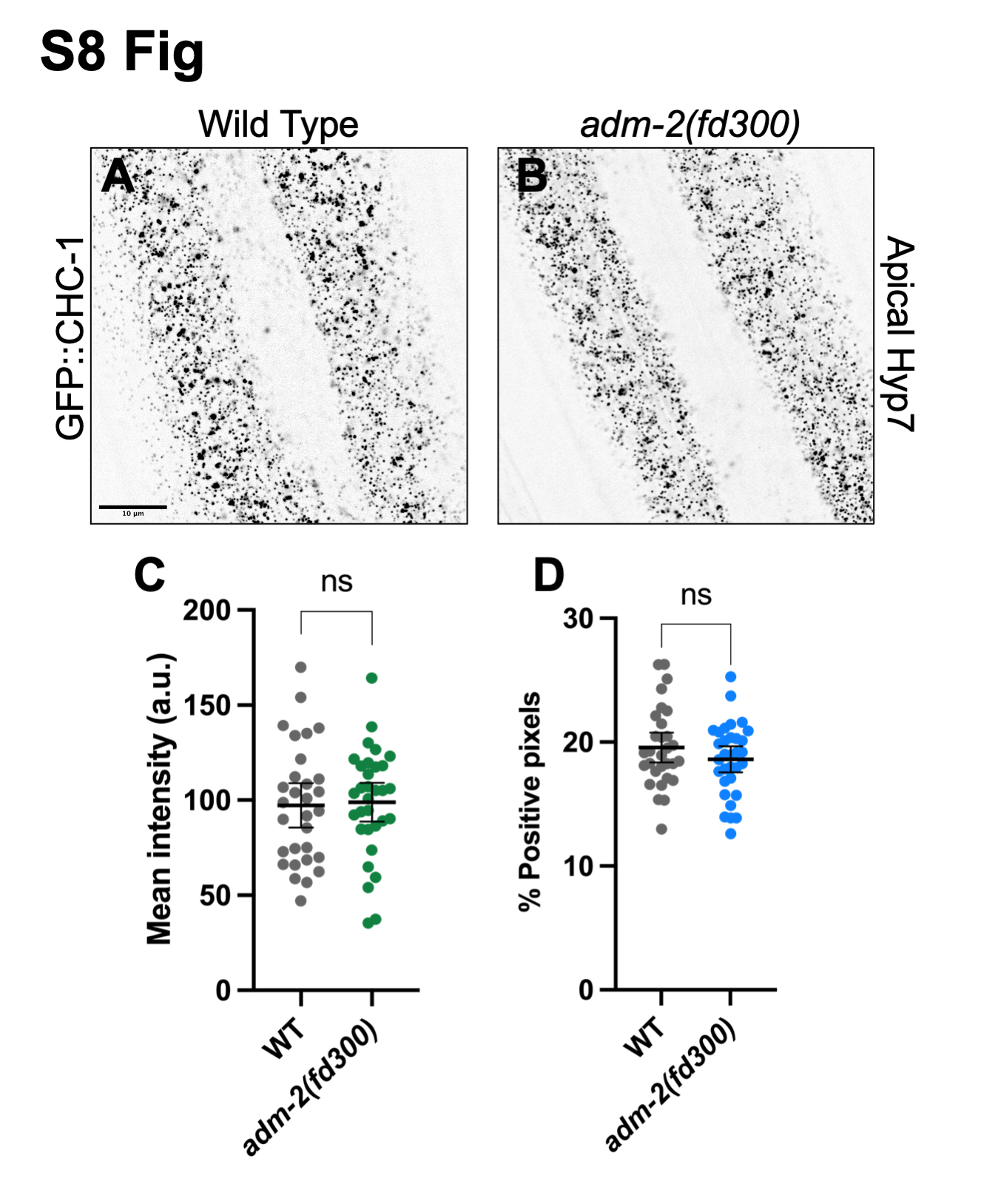

Supplement: S8 Fig — (A,B) Representative confocal images of GFP::CHC-1 expression in the apical hyp7 region of the hypodermis in wild-type (A) and adm-2(fd300) null mutant (B) day-1 adult worms. Bar in A = 10 μm (for A, B). (C, D) Dot plots showing GFP::CHC-1 mean intensity (a.u.) (C) and the percentage of GFP-positive pixels (D) within the apical plane for individual worms of the specified genotype. In C and D, group means along with 95% confidence intervals (error bars) are indicated. p-values were obtained by comparing means using an unpaired t-test. ns, p > 0.05. Raw data for this figure is provided in S1 File. (TIFF) [file pgen.1010249.s008.tiff]

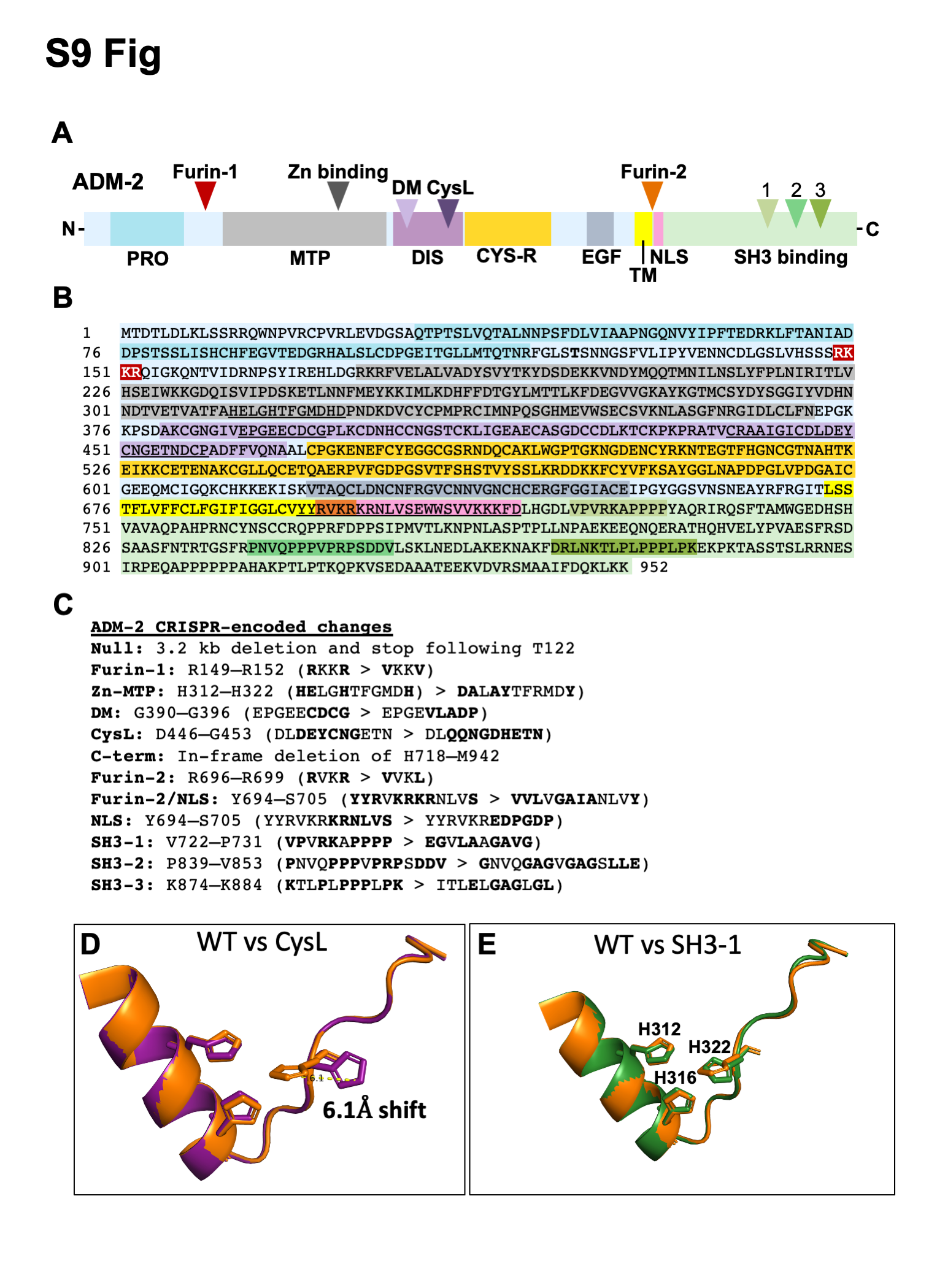

Supplement: S9 Fig — (A) Schematic representation of predicted protein domains within ADM-2. (B) Color-coded peptide sequence of ADM-2 corresponding to panel A. For additional details see S2 Fig. (C) Amino acid sequence change details of the ADM-2 variants generated using CRISPR methods. (D, E) Predicted three-dimensional protein structures (for amino acid region 307–328) of wild-type ADM-2 (orange) superimposed on modeled structures for CysL (D; violet), SH3-1(E; green) mutant proteins. Three conserved histidine residues (His312, His316, His322) of the Zn-metalloprotease domain are represented as sticks. A predicted 6.1-Å shift of the ‘tele’ nitrogen atom in the imidazole ring of His322 was predicted in the CysL variant. (TIFF) [file pgen.1010249.s009.tiff]
