# Supplementary material for: An unexpected role for the conserved ADAM-family metalloprotease ADM-2 in Caenorhabditis elegans molting
Source: PLoS Genet. 2022 May 31;18(5):e1010249. doi: 10.1371/journal.pgen.1010249 (PMC9187072; doi:10.1371/journal.pgen.1010249)
Supplement: S2 Fig — Peptide alignment of C. elegans ADM-2 with human meltrin family members (ADAM9/12/19/33). Predicted domains of ADM-2 are color coded. NLS, nuclear localization domain. (PDF) [file pgen.1010249.s002.pdf]

S2 Fig. Alignment of *C. elegans* ADM-2 with human ADAM9/12/19/33

```
ADM-2      1 MTDTLDL------------------------------------------KLSSR----RQ
ADAM9      1 MGSGARFPSGTLRVRWLLLLGLVGPVLG---AAR-PGFQQTS-------HLSSYEII-TP
ADAM12     1 MAAR-PLPVSPA--RALL-LALAGALLAPCEARGVSLWNQGRADEVVSASVGSG----DL
ADAM19     1 MPGG----AGAA--RLCL-LAFALQPLRPRAARE-PGWTRGSE--EGSPKLQHE-LIIPQ
ADAM33     1 ------------------------------------------------------------

ADM-2     15 WNPVRCPVRLEVDGSAQTPTSL--VQTALNNPSFDLVIAAPNGQNVYI-PFTEDRKLFTA
ADAM9     49 WRLTRERRE------APRP-YSKQVSYVIQAEGKEHIIHLERNKDLLPEDFVVYTYNKEG
ADAM12    53 WIPVKSFDS------KNHP-EV--LNIRLQRESKELIINLERNEGLIASSFTETHYLQDG
ADAM19    50 WKTSESPVR------EKHP-LK--AELRVMAEGRELILDLEKNEQLFAPSYTETHYTSSG
ADAM33     1 ----------------SKP-DM--GLVALEAEGQELLIELEKNHRLLAPGYIETHYGPDG

ADM-2     72 NIADDPSTS---SLISHCHFEGVTEDG-RHALSLCDPGEITGLLMTQTNRFGLSTSNNGS
ADAM9    102 --TLITDHP---NIQNHCHYRGYVEGVHNSSIALSDCFGLRGLLHL----------ENAS
ADAM12   104 --TDVSLARNYTVILGHCYYHGHVRGYSDSAVSLSTCSGLRGLIVF----------ENES
ADAM19   101 --NPQTTTR---KLEDHCFYHGTVRETELSSVTLSTCRGIRGLITVS---------SNLS
ADAM33    42 --QPVVLAP---NHTDHCHYQGRVRGFPDSWVVLCTCSGMSGLITLS---------RNAS
                                                   Cysteine switch
                                          Furin-1 (R149-R152)
ADM-2    128 FVLIPYVEN--NCDLGSLVHSSSRKKRQIGKQ----------------NT-VIDRNPS--
ADAM9    147 YGIEPLQNS--S-H-FE--HIIYRMDDVYKEPLKCGVSNKDIEKETAKDEEEEPPSMTQL
ADAM12   152 YVLEPMKSA--T---NR--YKLFPAKKLKSVRGSCGSHHNTP-NLAAKNV-FPPPSQTWA
ADAM19   147 YVIEPLPDS--K---GQ--HLIYRSEHLKPPPGNCGFEHSKPTTRDWALQ-FTQQTKKRP
ADAM33    88 YYLRPWPPRGSKDF-ST--HEIFRMEQLLTWKGTCG--HRDPGNKACMTS-LPGGPQS--

                        Protease domain (R177-P373)
ADM-2    167 -YIREHLDGRKRFVELALVADYSVYTKYDSDEKKVNDYMQQTMNILNSLYFPLNIRITLV
ADAM9    201 LRRRRAVLPQTRYVELFIVVDKERYDMMGRNQTAVREEMILLANYLDSMYIMLNIRIVLV
ADAM12   203 RRHKRETLKATKYVELVIVADNREFQRQGKDLEKVKQRLIEIANHVDKFYRPLNIRIVLV
ADAM19   199 RRMKREDLNSMKYVELYLVADYLEFQKNRRDQDATKHKLIEIANYVDKFYRSLNIRIALV
ADAM33   140 -RGRREARRTRKYLELYIVADHTLFLTRHRNLNHTKQRLLEVANYVDQLLRTLDIQVALT

ADM-2    226 HSEIWKKGDQISVIPDSKETLNNFMEYKKI-MLKDHFFDTGYLMTTLKFDEGVVGKAYKG
ADAM9    261 GLEIWTNGNLINIVGGAGDVLGNFVQWREKFLITRRRHDSAQLVLKKGFG-GTAGMAFVG
ADAM12   263 GVEVWNDMDKCSVSQDPFTSLHEFLDWRKMKLLPRKSHDNAQLVSGVYFQGTTIGMAPIM
ADAM19   259 GLEVWTHGNMCEVSENPYSTLWSFLSWRRK-LLAQKYHDNAQLITGMSFHGTTIGLAPLM
ADAM33   199 GLEVWTERDRSRVTQDANATLWAFLQWRRG-LWAQRPHDSAQLLTGRAFQGATVGLAPVE

                                      Zn binding (H312-H322)
ADM-2    285 TMCSYDYSGGIYVDHNNDTVETVATFAHELGHTFGMDHDPND-KDVCYCP--MPRCIMNP
ADAM9    320 TVCSRSHAGGINVFGQITVETFASIVAHELGHNLGMNHDDGR-D--CSCG--AKSCIMNS
ADAM12   323 SMCTADQSGGIVMDHSDNPLGAAVTLAHELGHNFGMNHDTLDRGCSCQMAVEKGGCIMNA
ADAM19   318 AMCSVYQSGGVNMDHSENAIGVAATMAHEMGHNFGMTHDSAD-C--CSASAADGGCIMAA
ADAM33   258 GMCRAESSGGVSTDHSELPIGAAATMAHEIGHSLGLSHDPDG-C-CVEAAAESGGCVMAA
                                         Disintegrin domain (A380-A467)
                                           Disintegrin motif (E388-G396)
ADM-2    342 QSGH--MEVWSECSVKNLASGFNRGIDLCLFNEPGKK--PSDAKCGNGIVEPGEECDCGP
ADAM9    375 GAS--GSRNFSSCSAEDFEKLTLNKGGNCLLNIPKPDEAYSAPSCGNKLVDAGEECDCGT
ADAM12   383 STGYPFPMVFSSCSRKDLETSLEKGMGVCLFNLPEVRESFGGQKCGNRFVEEGEECDCGE
ADAM19   375 ATGHPFPKVFNGCNRRELDRYLQSGGGMCLSNMPDTRMLYGGRRCGNGYLEDGEECDCGE
ADAM33   316 ATGHPFPRVFSACSRRQLRAFFRKGGGACLSNAPDPGLPVPPALCGNGFVEAGEECDCGP

                                             Cysteine loop (C438-P459)
ADM-2    398 -LKCD-NHCCNGSTCKLIGEAECASGDCCDLKTCKPKPRATVCRAAIGICDLDEYCNGET
ADAM9    433 PKECELDPCCEGSTCKLKSFAECAYGDCCK--DCRFLPGGTLCRGKTSECDVPEYCNGSS
ADAM12   443 PEECM-NRCCNATTCTLKPDAVCAHGLCCE--DCQLKPAGTACRDSSNSCDLPEFCTGAS
ADAM19   435 EEECN-NPCCNASNCTLRPGAECAHGSCCH--QCKLLAPGTLCREQARQCDLPEFCTGKS
ADAM33   376 GQECR-DLCCFAHNCSLRPGAQCAHGDCCV--RCLLKPAGALCRQAMGDCDLPEFCTGTS


                          Cysteine-rich domain (C470-C611)
ADM-2    456 NDCEADFFVQNAALCPGKENEFCYEGGCGSRNDQCAKLWGPTGKNGDENCYR-KNTEGTF
ADAM9    491 QFCQPDVFIQNGYPCQNN-KAYCYNGMCQYYDAQCQVIFGSKAKAAPKDCFIEVNSKGDR
```

```
ADAM12   500  PHCPANVYLHDGHSCQDV-DGYCYNGICQTHEQQCVTLWGPGAKPAPGICFERVNSAGDP
ADAM19   492  PHCPTNFYQMDGTPCEGG-QAYCYNGMCLTYQEQCQQLWGPGARPAPDLCFEKVNVAGDT
ADAM33   433  SHCPPDVYLLDGSPCARG-SGYCWDGACPTLEQQCQQLWGPGSHPAPEACFQVVNSAGDA

ADM-2    515  HGNCGTNAHTKEIKKCETENAKCGLLQCETQAERPVFGDPGSVTFSHSTVYS-SLKRDDK
ADAM9    550  FGNCGFS-G-NEYKKCATGNALCGKLQCENVQEIPVFGI-V--P---AIIQT-PSRGTKC
ADAM12   559  YGNCGKVSK-SSFAKCEMRDAKCGKIQCQGGASRPVIGT-NAVSIE-TNIPLQQGGRILC
ADAM19   551  FGNCGKDMN-GEHRKCNMRDAKCGKIQCQSSEARPLE-S-NAVPID-TTIIM-NGRQIQC
ADAM33   492  HGNCGQDSE-GHFLPCAGRDALCGKLQCQGGKPSLLA-P-HMVPVD-STVHL-DGQEVTC

ADM-2    574  KFCYVFKSAYG----GLNAPDPGLVPDGAICGEEQMCIGQKCHKKEKISKVTA-QCLDNC
ADAM9    601  WGVD----FQL----GSDVPDPGMVNEGTKCGAGKICRNFQCVDASVL-N-YDCDVQKKC
ADAM12   616  RGTH----VYL----GDDMPDPGLVLAGTKCADGKICLNRQCQNISVF-G-VH-ECAMQC
ADAM19   606  RGTH----VYRGPEEEGDMLDPGLVMTGTKCGYNHICFEGQCRNTSFF-E-TE-GCGKKC
ADAM33   547  RGAL----ALPS--AQLDLLGLGLVEPGTQCGPRMVCQSRRCRKNAFQ-E-LQ-RCLTAC

              EGF-like(V620-V652)            Transmembrane Domain(L763-Y695)
ADM-2    629  NFRGVCNNVGNCHCERGFGGIACEIPGYGGSVNSNEAYRFRGITLSSTFLVFFCL-FGIF
ADAM9    651  HGHGVCNSNKNCHCENGWAPPNCETKGYGGSVDSGPTYNEMNTALRDGLLVFFFL-I--V
ADAM12   665  HGRGVCNNRKNCHCEAHWAPPFCDKFGFGGSTDSGPIRQADNQGLTIGILV ILC-L--L
ADAM19   659  NGHGVCNNNQNCHCLPGWAPPFCNTPGHGGSIDSGPMPPESVGPVVAGVLVAILV-L--A
ADAM33   598  HSHGVCNSNHNCHCAPGWAPPFCDKPGFGGSMDSGPVQAENHDTFLLAMLLSVLLPL--L

                  Furin-2(R696-R699) NLS(R696-D716)  SH3 binding-1
ADM-2    688  IGG---LCVYYRVKRKRNLV-SEWWSVVKKKFDLHGDLVPVRKAPPPPYAQRIRQSFTAM
ADAM9    708  PLIVCAIFIF--IKRD-QLW----RSY---------------------FRKKRSQTYE--
ADAM12   722  AAG---FVVY--LKRK-TLI----RLL---------------------FTNKKT-TIE--
ADAM19   716  VLM---LMYY--CCRQNNKLGQLKPSA---------------------LPSKLRQQFSCP
ADAM33   656  PGA---GLAW--CCYR-LP-----------------------------------------

ADM-2    744  ------WGEDHSH--VA----VAQPAHPRNCYN--SCCRQP-PRFDPPSIPMVTLKNPNL
ADAM9    738  -----SDGKNQAN---------------------------P-SR-QPGSVP-RHVSPVTP
ADAM12   748  ----KL---RCVRPSRPPRGFQPCQAHLGHLGK--GLMRKPPDSYPPKDNPRRLLQCQNV
ADAM19   750  FRVSQNSGTGHANPTFK----LQTPQGKRKVINTPEILRKP-SQPPPR-PPPDYLRGGSP
ADAM33   669  ------------------------GAHLQRCSW--GCRRDP-ACSGPKDGPHRD------

ADM-2    789  --ASPTPLLNPAEKEEQNQERA------------THQHVELYPVAESFRSDSAASFNTRT
ADAM9    763  --PREVP---------IYANRFAVP----------TYAAKQPQQFPSRPPPPQPKVSS-Q
ADAM12   799  DISRPLNGLNVP--QPQSTQRVLPP----------LHRAPRAPSVPARPLPAKPALRQAQ
ADAM19   804  --PAPLP---------AHLSRAARNSPGPGSQIERTESSRR--PPPSRPIPPAPNCIVSQ
ADAM33   696  ---HPLGG---------------------------VHPMELGPTATGQPWPLDPENSH-E

                  SH3 binding-2                   SH3 binding-3
ADM-2    835  GSFRPNVQPPPVPRPSDDV--LSKLNEDLAKEKNAKFDRLNKTLPLPPPLPKEKPKTASS
ADAM9    801  GNL---I--PARPAPAPPLYSSLT------------------------------------
ADAM12   847  GTCKPNP--PQKPLPADPLARTTRLTHALARTPG-QWETGLRLAPL---------------
ADAM19   851  DFSRPRP--PQKALPANPVPGRRSLPRPGG-----------ASPL---------------
ADAM33   725  PSS---H--PEKPLPAV-------------------------------------------

                        SH3 binding(?)
ADM-2    893  TSLRRNESIRPEQAPPPPPPAHAKPT--LPTKQPKVSEDAAATEEKVDVRSMAA------
ADAM9         ------------------------------------------------------------
ADAM12   890  ----------------R----------------PA-------------------------
ADAM19   883  ----------------RPPGAGPQQSRPLAALAPKVSPREA-LKVKAGTRGLQGGRCRVE
ADAM33   737  ----------------S----------------PD-------------------------

ADM-2    945  ---------IF-----DQKLKK---------
ADAM9         -------------------------------
ADAM12   893  -----------PQYP-HQVPRSTHTAYIK--
```

```
ADAM19  926 KTKQFMLLVVWTELP-EQKPRAKHSCFLVPA
ADAM33  740 -----------PQADQVQMPRSCLW------
```
