# Supplementary material for: An unexpected role for the conserved ADAM-family metalloprotease ADM-2 in Caenorhabditis elegans molting
Source: PLoS Genet. 2022 May 31;18(5):e1010249. doi: 10.1371/journal.pgen.1010249 (PMC9187072; doi:10.1371/journal.pgen.1010249)
Supplement: S1 Table — (PDF) [file pgen.1010249.s010.pdf]

S1 Table. Strains used in this study

Strain	Genotype
RT3402	*pw17(gfp::chc-1)*
WY1145	*nekl-2(fd81); nekl-3(gk894345); fdEx286 (pDF153(nekl-3 (+)); pTG96(sur-5::GFP))*
WY1208	*nekl-2(fd81); nekl-3(gk894345) adm-2(fd130)*
WY1232	*nekl-2(fd81); nekl-3(gk894345); fdEx186* (*nekl-3*$^{+}$ + SUR-5::GFP); *fdEx197* (SUR-5::RFP)]
WY1279	*nekl-2(fd81); nekl-3(gk894345) adm-2(fd163)*
WY1347	*nekl-2(fd81); nekl-3(gk894345) adm-2(fd208)*
WY1342	*nekl-2(fd81); pwIs528(GFP::CHC-1); nekl-3(gk894345) adm-2(fd130); fdEx286*
WY1386	*nekl-2(fd81); pwIs528(GFP::CHC-1); nekl-3(gk894345) adm-2(fd130); fdEx286; fdEx315 (adm-2 fosmid mix; WRM0620dD12, WRM0632aG02, and WRM0610cA04)* [Line #1]
WY1388	*nekl-2(fd81); pwIs528(GFP::CHC-1); nekl-3(gk894345) adm-2(fd130); fdEx286; fdEx356 (adm-2 fosmid mix; WRM0620dD12, WRM0632aG02, and WRM0610cA04)* [Line #2]
WY1428	*nekl-2(fd81); nekl-3(gk894345) adm-2(fd228)* [large deletion]
WY1429	*nekl-2(fd81); nekl-3(gk894345) adm-2(fd229)* [large deletion]
WY1430	*nekl-2(fd81); nekl-3(gk894345) adm-2(fd230)* [large deletion]
WY1435	*adm-2(fd235)* [large deletion]
WY1436	*adm-2(fd236)* [large deletion]
WY1437	*adm-2(fd237)* [large deletion]
WY1605	*pw17(gfp::chc-1); adm-2(fd300)*
WY1640	*nekl-2(fd91); adm-2(fd313); fdEx278* [pDF166 (*nekl-2* genomic) + pTG96 (SUR-5::GFP)]
WY1643	*nekl-3(sv3) adm-2(fd316); mnEx174* [F19H6 (*nekl-3* genomic) + pTG96 (SUR-5::GFP)]
WY1644	*mlt-4(sv9); adm-2(fd317) mlt-4(sv9); mnEx173* [ZC15 (*mlt-4* genomic) + pTG96 (SUR-5::GFP)]
WY1562	*eqIs1(lrp-1::gfp); ieSi57(peft-3::mRuby::tir-1); pw29(nekl-3::aid)*
WY1654	*eqIs1(lrp-1::gfp); fcho-1(ox477::unc-119(+)); ieSi57(peft-3::mRuby::tir-1); pw29(nekl-3::aid)*
WY1655	*eqIs1(lrp-1::gfp); adm-2(fd318); ieSi57(peft-3::mRuby::tir-1); pw29(nekl-3::aid)*
WY1656	*eqIs1(lrp-1::gfp); adm-2(fd300)*
LH191	*eqIs1(lrp-1::gfp); rrf-3(pk1426)*
WY1657	*adm-2(fd300)*
PHX2391	*adm-2::eGFP (syb2391)*
PHX1722	*adm-2::mScarlet (syb1722)*
WY1664	*pw17(gfp::chc-1); adm-2::mScarlet (syb1722)*
WY1675	*adm-2::mScarlet (syb1722); pwSi125[phyp7::NeonGreen::hgrs-1]*
WY1820	*N2; fdEx373*
WY1821	*N2; fdEx374*
WY1822	*N2; fdEx375*
WY1833	*N2; fdEx382*
WY1841	*eqIs1(lrp-1::gfp); fdEx382*
WY1893	*pw27(nekl-2::aid); ieSi10(phyp7::BFP::tir-1); adm-2::mScarlet (syb1722)*
WY1897	*ieSi10(phyp7::BFP::tir-1); pw29(nekl-3::aid) adm-2::mScarlet (syb1722)*
WY1431	*nekl-2(fd81); nekl-3(gk894345) adm-2(fd231)*

WY1513	*nekl-2(fd81); nekl-3(gk894345) adm-2(fd243)*
WY1585	*nekl-2(fd81); nekl-3(gk894345) adm-2(fd288)*
WY1518	*nekl-2(fd81); nekl-3(gk894345) adm-2(fd248)*
WY1522	*nekl-2(fd81); nekl-3(gk894345) adm-2(fd252)*
WY1527	*nekl-2(fd81); nekl-3(gk894345) adm-2(fd257)*
WY1676	*nekl-2(fd81); nekl-3(gk894345) adm-2(fd324)*
WY1669	*nekl-2(fd81); nekl-3(gk894345) adm-2(fd322)*
WY1589	*nekl-2(fd81); nekl-3(gk894345) adm-2(fd292)*
WY1590	*nekl-2(fd81); nekl-3(gk894345) adm-2(fd292)*
WY1637	*nekl-2(fd81); nekl-3(gk894345) adm-2(fd310)*
WY1638	*nekl-2(fd81); nekl-3(gk894345) adm-2(fd311)*
WY1639	*nekl-2(fd81); nekl-3(gk894345) adm-2(fd312)*
WY1678	*nekl-2(fd81); nekl-3(gk894345) adm-2(fd326)*
WY1676	*nekl-2(fd81); nekl-3(gk894345) adm-2(fd324)*
WY1677	*nekl-2(fd81); nekl-3(gk894345) adm-2(fd325)*
WY1971	*eqIs1(lrp-1::gfp); adm-2(fd391)*
PHX4599	*lrp-1::mScarlet (syb4599)*
WY1959	*lrp-1::mScarlet (syb4599); adm-2(fd300)*
WY1958	*eqIs1(lrp-1::gfp); fdEx400*
WY1957	*eqIs1(lrp-1::gfp); fdEx396*
WY1929	*eqIs1(lrp-1::gfp); ; adm-2::mScarlet (syb1722)*
