# Supplementary material for: An unexpected role for the conserved ADAM-family metalloprotease ADM-2 in Caenorhabditis elegans molting
Source: PLoS Genet. 2022 May 31;18(5):e1010249. doi: 10.1371/journal.pgen.1010249 (PMC9187072; doi:10.1371/journal.pgen.1010249)
Supplement: S3 File — (PDF) [file pgen.1010249.s013.pdf]

File3: Detailed sequencing data for *adm-2* alleles

Mutatagenesis Alleles

STOP codon

WY1208 *nekl-2(fd81); nekl-3(gk894345) adm-2(fd130)*

GAGTTCTGTTACGAAGGTGGCTGTGGTAGTCGAAACGATCAATGCGCAAAGTTATGAGGTCCTA
CTGGGAAAAATGGCGATGAGAACTGCTATCGTAAAAACACTGAAGGAACCTTTCATGGAAACTG

NOTES: Premature stop Trp>Stop [TGG>TG**A**].

WY1279 *nekl-2(fd81); nekl-3(gk894345) adm-2(fd163)*

TCCGTCATTCGACCTGGTGATTGCCGCGCCAAACGGTCAAAATGTGTACATACCGTTTACGGAA
GATAGATAGGTTTGAAACTGTGAAAATAAATTTTTAACAAAAATGTTGAGAATGGTGAACAAAA

Notes: 5′ splice donor sequence intron-1 (GT>AT)

CRISPR Alleles

Mutation changes

STOP codon

WY1428 *nekl-2(fd81); nekl-3(gk894345) adm-2(fd228)*

TTAAAATCATATTATTTAAATTAATGCAAATTGTTTTTTTTTCAAAAATCACGGGACTTCTTAT
GACGCAAACCAATCGGTTCGGTCTTTCCACCACAGCTGGTCAGCAGGGGCTGGGGTAGTCGAAA
CGATCAAGGCGCAAAGTTATGGGGTCCTACTGGGAAAAATGGCGATGAAAACTGCTATCGTAAA

NOTES: Large deletion results in premature stop after aa 130.

WY1429 *nekl-2(fd81); nekl-3(gk894345) adm-2(fd229)*

CTTAAAATCATATTATTTAAATTAATGCAAATTGTTTTTTTTTCAGAAATCACGGGACTTCTTA
TGACGCAAACCAATCGGTTCGGTCTTTCCACCTCCTAGGCTGTTGGTCTGAAGCCATGTGAAGC
TCCGCAACACTTCCGCAGCTGCGCTGAAGCAACAGGGGGNNGGGGTAGTCGAAACGATCAATGC

NOTES: Large deletion results in premature stop after aa 123.

WY1430 *nekl-2(fd81); nekl-3(gk894345) adm-2(fd230)*

AAATCATATTATTTAAATTAATGCAAATTGTTTTTTTTTCAAAAATCACGGGACTTCTTATGAC
GCAAACCAATCGGTTCGGTCTTTCCCCTGTTTGTCCANTGTTATGACTGGGNTAGTCGAAACGA
TCAAGGCGCAAAGTTATGGGGTCCTACTGGGAAAAATGGCGATAANAACTGCTATCGTAAAAAC

NOTES: Large deletion results in premature stop after aa 127.

WY1435 *adm-2(fd235)*

AAAACAAAAAAAAATTTTCAGTCATCAAATCAACGCAAATTTTTTTAAAAGTCGAACATTATAGA
ACTTAAAATCATATTATTTAAATTAATGCAAATTGTTTTTTTTTCAAAAATCACGGGACTTCTT
ATGACGCAAACCAATCGGTTCGGTCTTTCCACCTGGAGGGGGTTGGGGAAGTCGAAACGATCAA
TGCGCAAAGTTATGGGGTCCTACTGGGAAAAATGGCGATGAAAACTGCTATCGTAAAAACACTG

Notes: Large deletion results in premature stop after aa 145.

WY1436 *adm-2(fd236)*

AAATCATATTATTTAAATTAATGCAAATTGTTTTTTTTTCAAAAATCACGGGACTTCTTATGAC
GCAAACCAATCGGTTCTGGGGTAGTCCAACAGCGTAACAACAGCTGTGAAATGAAAATGACTGG
AAACCGTACCGCAGGCGGGGCCTAAGGGTAGCGGACAACAGCTGGGGTAGTCGAAACGATCAAT

Notes: Large deletion results in premature stop after aa 124.

WY1437 (*fd237*)
ACTTAAAATCATATTATTTAAATTAATGCAAATTGTTTTTTTTCAAAAATCACGGACTTCTT
ATGACGCAAACCAATCGGTTCGGTCTTTCGACTTACTAGGTAGTCAAAACGATCAATGCGCAAA
GTTATGGGGTCCTACTGGGAAAAATGGCGATGAAAACTGCTATCGTAAAAACACTGAAGGAACC
Notes: Large deletion results in premature stop after aa 123.

In-frame deleted region between blue and green
WY1431 *nekl-2(fd81); nekl-3(gk894345) adm-2(fd231)*
TCACTATTTTTATTATCATTTCTTGCAGATGGTGGAGTGTTGTCAAGAAGAAGTTTGATTTACT
CGAGGCTGCTATATTTGATCAAAAGCTCAAAAAATAACCGGATTCTCTCAATCTTAAATTTCAA
ATTTTCAATTTAAAATTTCTGATGTTCNTATTAAAACCTTTCCCTNATCCTCAAAAACTNGNCA
Notes: Removes C-terminal coding sequences from H718 to M942.

WY1513 *nekl-2(fd81); nekl-3(gk894345) adm-2(fd243)*
WY1971 *eqIs1(lrp-1::gfp); adm-2(fd391)*
AACACATAAGAGAAATTCCATGTTTTCAGGACCACAACAATGATACGGTCGAAACAGTTGCGAC
TTTTGCTGACGCACTCGCCTATACATTTCGAATGGATTATGACCCGAATGACAAGGACGTGTGC
TACTGTCCAATGCCACGATGCATCATGANTCCCCAATCTGGGCATATGGAAGTTTGG
Notes: Zn-binding [H312–H322 (**HE**L**GH**TF**G**MD**H**) to **DA**L**AY**TF**R**MD**Y**]

WY1518 *nekl-2(fd81); nekl-3(gk894345) adm-2(fd248)*
ACATTTTAATCGAGCTGATCACTATTTTTATTATCATTTCTTGCAGATGGTGGAGTGTTGTCAA
GAAGAAGTTTGATTTACACGGAGACCTCGAGGGCGTGCTGGCGGCGGGCGCGGTGGGCTATGCT
CAACGAATTCGGTTGGTTTTTTCATTCACATTTCCTTACCAAAATTCCAACTTTACAGCCAAAG
Notes: SH3-1 [V722–P731 (**VP**V**RK**A**PPPP**) to **EG**V**LA**A**GAVG**]

WY1522 *nekl-2(fd81); nekl-3(gk894345) adm-2(fd252)*
AGGCTTTCAATCTTTTCTCAAATTTCTTACTGTTTATGATTTTAGGCTTCCTTCAACACGCGCA
CTGGATCCTTCCGTGGCAACGTGCAGGGCGCGGGCGTGGGCGCGGGCAGCCTGCTCGAGCTGAG
TAAACTGAATGAGGACCTGGCAAAGGAGAAAAATGCGAAATTTGATCGACTGTGAGAATATAGT
Notes: SH3-2 [P839–V853 (**P**NVQ**PPP**V**PRP**S**DDV**) to **G**NVQ**GAG**V**GAG**S**LLE**]

WY1527 *nekl-2(fd81); nekl-3(gk894345) adm-2(fd257)*
AGGAGAAAAATGCGAAATTTGATCGACTGTGAGAATATAGTTGTTTATTTCATCAAAAGTCACG
TTTTTGCAGAAACATTACCCTCGAGCTGGGCGCGGGCCTGGGCCTGGAAAAACCGAAAACTGCA
AGTTCTACGAGCCTGAGAAGAAATGAGAGTATACGACCCGAGCAGGCACCACCACCACCTCCAC
Notes: SH3-2 [K874–K884 (**K**TL**P**L**PPP**L**PK**) to **I**TL**E**L**GAG**L**GLB**]

WY1585 *nekl-2(fd81); nekl-3(gk894345) adm-2(fd288)*
CCTACTTGTTCAAAACTAGCAATGTTCATCCAGCTGTCTATGTCGTATCAATGTATAGTTAAAA
AATAATCAACACCACATTTTTACAGATCTCGGATCCCTAGTCCATTCCTCGTCTGTTAAAAAAG
TTCAGATTGGAAAACAGAATACTGTGATAGATCGTAATCCATCATACATCAGGGAACATCTGGA
Notes: Furin-1 [R149–R152 (**R**KK**R**) to **V**KK**V**].

WY1589 *nekl-2(fd81); nekl-3(gk894345) adm-2(fd310)*
TATAATTCAGAATTACACTGTCGTCGACATTCTTGGTATTTTTCTGCTTGTTTGGAATTTTTAT
CGGTGGTCTTTGTGTTTATTACGTCGTTAAACTTAAGCGAAATTTGGTCTCGGAGTAAGTTTTT
GAAATGTATGAGTTCTTTGGAAATTATACTTTAACTCAAATTTATCTAAAAAGTAACTAAAATA
Notes: Furin-2 [R696–R699 (**R**VK**R** to **V**VK**L**)].

WY1637 *nekl-2(fd81); nekl-3(gk894345) adm-2(fd292)*
TATAATTCAGAATTACACTGTCGTCGACATTCTTGGTATTTTTCTGCTTGTTTGGAATTTTTAT
CGGTGGTCTTTGTGTTGTGGTGCTCGTTGGTGCTATTGCGAATTTGGTCTACGAGTAAGTTTTT
GAAATGTATGAGTTCTTTGGAAATTATACTTTAACTCAAATTTATCTAAAAAGTAACTAAAATA
Notes: Furin-2/NLS [Y694–V704 (**YYR**V**KRKR**NL**V** to **VVL**V**GAIA**NLV**Y**)].

WY1637 *nekl-2(fd81); nekl-3(gk894345) adm-2(fd326)*
AATTACACTGTCGTCGACATTCTTGGTATTTTTCTGCTTGTTTGGAATTTTTATCGGTGGTCTT
TGTGTTTATTACAGAGTTAAACGGGAGGATCCCGGGGATCCAGAGTAAGTTTTTGAAATGTATG
AGTTCTTTGGAAATTATACTTTAACTCAAATTTATCTAAAAAGTAACTAAAATATATCTAAAAA
Notes: NLS [K700–S705 (**KRNLVS** > **EDPGDP**)].

Start ATG
WY1603 *pw17(gfp::chc-1); adm-2(fd298)*
TCTCATTTCTCTAAACAGTTTTGTTTCGTTTCTTATACTTTTCTGTATGCACTTCACGTGCTCT
GCATAGCTAATTTTCTTAAATTTCAGTATGCATAAACAATGACCGTTATTAATTGTTAAGAACA
AATGAAACATGTCACAAGGATTTAAAGTTTGAGTCTGCTAGTAAAAAGCGCGTTTACAGCAATG
Notes: ~7.4-kb deletion + 5 bp insertion.

WY1604 *pw17(gfp::chc-1); adm-2(fd299)*
TCTCATTTCTCTAAACAGTTTTGTTTCGTTTCTTATACTTTTCTGTATGCACTTCACGTGCTCT
GCATAGCTAATTTTCTTAAATTTCAGTATGCATAAACAATGAGCTATATTTGATCAAAAGCTCA
AAAAATAACCGGATTCTCTCAATCTTAAATTTCAAATTTTCAATTTAAAATTTCTGATGTTCAT
Notes: ~7.4-kb deletion + 3 bp insertion.

WY1605 *pw17(gfp::chc-1); adm-2(fd300)*
TCTAAACAGTTTTGTTTCGTTTCTTATACTTTTCTGTATGCACTTCACGTGCTCTGCATAGCTA
ATTTTCTTAAATTTCAGTATGCATAAACAATGACCGACTCCACTCCATGGCTGCTATATTTGAT
CAAAAGCTCAAAAAATAACCGGATTCTCTCAATCTTAAATTTCAAATTTTCAATTTAAAATTTC
Notes: ~7.4-kb deletion + 4 bp insertion.

WY1640 *nekl-2(fd91); adm-2(fd313); fdEx278* [pDF166 (*nekl-2* genomic) + pTG96 (SUR-5::GFP)]
CTGAAAATTGGCTCTCATTTCTCTAAACAGTTTTGTTTCGTTTCTTATACTTTTCTGTATGCAC
TTCACGTGCTCTGCATAGCTAATTTTCTTAAATTTCAGTATGCATAAACAATGACATGTTTTTG
GGCAAAAAATTCCAGCATTTTTTGTAGATTAAACCGTGATGGGACAACCTGGCACCACGTGCAA
TATTTGAACATTTTTAAATACTATCATCGCATTAGAAATCTGATAGGTTAAAAATAAAAAAACA
Notes: ~7.4-kb deletion.

WY1643 *nekl-3(sv3) adm-2(fd316); mnEx174* [F19H6 (*nekl-3* genomic) + pTG96 (SUR-5::GFP)]
TCNCTGAAAATTGGCTCTCATTTCTCTAAACAGTTTTGTTTCGTTTCTTATACTTTTCTGTATG
CACTTCACGTGCTCTGCATAGCTAATTTTCTTAAATTTCAGTATGCATAAACAATCAAACAATG
ATCAAAACAATGATCAAAAGCTCAAAAAATAACCGGATTCTCTCAATCTTAAATTTCAAATTTT
CAATTTAAAATTTCTGATGTTCATATTAAAACCTTTCCCTGATCCTCAAAAACTCGTCAAAAAC
Notes: ~7.4-kb deletion + 19 bp insertion.

WY1644 *mlt-4(sv9); adm-2(fd317) mlt-4(sv9); mnEx173* [ZC15 (*mlt-4* genomic) + pTG96 (SUR-5::GFP)]
GAAAATTGGCTCTCATTTCTCTAAACAGTTTTGTTTCGTTTCTTATACTTTTCTGTATGCACTT
CACGTGCTCTGCATAGCTAATTTTCTTAAATTTCAGTATGCATAAACAATGACCGATGATTTGA
ACATTTTTAAATACTATCATCGCATTAGAAATCTGATAGGTTAAAAATAAAAAAACAAGCGCTA
Notes: ~7.4-kb deletion.

WY1646 *eqIs1(lrp-1::gfp); adm-2(fd318); ieSi57(peft-3::mRuby::tir-1); pw29(nekl-3::aid)*
GAAAATTGGCTCTCATTTCTCTAAACAGTTTTGTTTCGTTTCTTATACTTTTCTGTATGCACTT
CACGTGCTCTGCATAGCTAATTTTCTTAAATTTCAGTATGCATAAACAATGACCGACATATTTG
ATCAAAAGCTCAAAAAATAACCGGATTCTCTCAATCTTAAATTTCAAATTTTCAATTTAAAATT
Notes: ~7.4-kb deletion.
