# Supplementary material for: An unexpected role for the conserved ADAM-family metalloprotease ADM-2 in Caenorhabditis elegans molting
Source: PLoS Genet. 2022 May 31;18(5):e1010249. doi: 10.1371/journal.pgen.1010249 (PMC9187072; doi:10.1371/journal.pgen.1010249)
Supplement: Attachment — Submitted filename: Joseph_Rebuttal_PLoSGen.pdf [file pgen.1010249.s014.pdf]

Response to reviewers:

We thank all three reviewers and the editor for their positive comments as well as their highly useful and instructive suggestions. Before addressing individual points, we thought it would be helpful to provide an overview of the substantive changes we've made. Since our first submission we have carried out a sizeable number of new experiments. Some of these were directly in response to questions raised by the reviewers; others were prompted by our own evolving discussions. All substantive changes connect to the final portion of the manuscript, which concerned the mechanism of LRP-1 regulation by ADM-2, as well as a broader question concerning the role(s) of *adm-2* in suppression and molting.

Our initial focus was to get western blots working for LRP-1::GFP, which was initially challenging in part due to its large (>500 kD) size. In fact, we were never successful in getting a clear LRP-1::GFP signal with a number of different anti-GFP antibodies, so we next turned to a newly generated ADM-2::mScarlet CRISPR strain (using an anti-RFP antibody), this time with success. As described in the manuscript (lines 317-348; Fig 6), although we obtained evidence that LRP-1 is likely to be proteolytically processed in worms, this processing does not appear to require ADM-2 activity. Specifically, loss of *adm-2* did not substantially alter levels of the processed LRP-1::mScarlet. We nevertheless we saw clear upregulation (~1.6-fold) of LRP-1::mScarlet in the epidermis of *adm-2* mutants, similar to our findings for LRP-1::GFP (Fig 5).

These results suggested that although ADM-2 may be a negative regulator of LRP-1, consistent with some reports in mammals, it apparently does so through a mechanism that is independent of its metalloproteinase (MTP) activity. To further solidify this unexpected result, we performed two additional experiments. (1) We found that an *adm-2* CRISPR variant targeting the MTP domain did NOT lead to an increase in LRP-1 levels, consistent with MTP-independent regulation of LRP-1 by ADM-2 (Fig 6B). (2) We overexpressed an MTP-defective ADM-2 variant and observed decreased levels of LRP-1, also consistent with MTP-independent regulation of LRP-1 by ADM-2 (Fig 6C,D; S7 Fig). Our data has led us to propose that ADM-2 regulates LRP-1 via a noncanonical mechanism, which could be argued to be a more interesting finding than our original model. Regardless, our current model is unquestionably more accurate. In addition, our data points to additional complexity in the suppression of *nekl* molting defects by *adm-2*. Namely, that multiple domains and functions likely contribute to the observed suppression (Fig 6E,F; S9 Fig, Lines 350-377).

One final point is that we generated an additional control for the ADM-2 heat shock experiments – one that was not requested by reviewers – but which we came up with post submission. Specifically, we generated a rol-6(gf)-marked array strain carrying empty vector (EV) heat-shock plasmids. As expected, LRP-1::GFP levels in this strain were not significantly different than what we had observed for the parental (non-array containing) strain, either before or after heat shock (Fig 6D; S7 Fig). Thus, our finding that HS-overexpression of wild-type ADM-2 (or MTP-defective ADM-2) decreases LRP-1 levels was solidified. What did surprise us, however, is that we observed some worms with molting defects after heat shocking this control strain. Although this combination of plasmids has been used quite extensively by the field, an effect on molting had not been previously reported, possibly because no one had looked

closely. In any case, this made us wary of interpreting molting defects using the rol-6(gf) + pHS array method and we have removed this data from the current manuscript (previous Fig 6 A,C,D). We think that this effect may be due to having HS elements juxtaposed with the rol-6(gf) collagen on the array because we did not observe molting defects in strains with a rol-6(gf) array following heat shock. We also attempted to overexpress ADM-2/ADM-2::GFP by heat shock using a sur-5::RFP array marker (instead of rol-6(gf), however these strains did not strongly induce ADM-2::GFP. The bottom line is that while we solidified our conclusion that ADM-2 overexpression reduces LRP-1 levels, we no longer feel comfortable claiming that ADM-2 overexpression causes molting defects and have thus removed this data.

Editorial decision:
The manuscript was fully evaluated at the editorial level and by independent peer reviewers. The reviewers appreciated the attention to an important topic but identified some concerns that we ask you address in a revised manuscript. Most comments likely can be addressed by rewriting, however reviewer 2 has made some suggestions for additional experiments that you may want to consider.

We therefore ask you to modify the manuscript according to the review recommendations. Your revisions should address the specific points made by each reviewer.

We thank the editor for their directions and note that we chose to address many of the comments/suggestions with further experimentation, including some experiments not explicitly suggested by reviewers.

Although most of these are discussed either above and/or below in the appropriate sections, we would note new S6 Fig, in which we tested an alternative hypothesis that *adm-2* mutations may suppress molting defects in part through induction of the *C. elegans* dauer pathway. This is based on findings from our recently published (2022) paper (PMID: 35038442), along with a previous report showing that a mutation in a different *C. elegans* ADAM family member (*gon-1*) could push worms towards the dauer fate (PMID: 26218657). In short, our result in S6 Fig did not support *adm-2* suppressing through a dauer-like mechanism. We have included text describing this experiment along with the rationale (Lines 282-288), which follows the section showing that loss of *adm-2* does not suppress *nekl*-associated trafficking defects.

Reviewer #1: This is a well written manuscript that describes fascinating work identifying the role of a conserved matrix metalloprotease, ADM-2, in roundworm molting, and provides evidence for a mechanism that includes endocytic trafficking that regulates ADM-2 activity and identify a candidate target, LRP-1, which may endocytose sterols that are used to create molting-specific steroid hormones that trigger molting.

While strong, the last piece of this puzzle, confirming their proposed target (LPR-1) is processed by their protease ADM-2, would strengthen support provided by their fluorescence imaging experiments for their model and, if possible, provide a satisfying conclusion. They have animals expressing the tagged target (LRP-1::GFP) in the relevant adm-2 backgrounds, and others

(whom they cite) have shown by western that metalloproteases including ADM-2-like ADAM-12 cleaves the LRP-1-related low-density lipoprotein receptor-related protein.

We strongly agreed with the reviewer's point. As a result, we followed up on their suggestion to test our model by westerns and other methods as described above (also see Fig 5, 6, S7). Admittedly, some of our findings have opened up a number of new questions regarding the mechanism(s) of *adm-2* suppression along with its effects on LRP-1, which are beyond the scope of the current study.

Minor points:
"LGX" is generally written "LG X".

Corrected.

S1 File: Is having more than one or two significant digits after the decimal (8 or 9 digits?) necessary and informative in the percent viability columns and the S7 Fig Average row?

Corrected.

S2 File:

The primer list sheet could use a little tidying up. SB3 doesn't include sequence information, sometimes a forward primer is designated with "F" and sometimes "fwd", and the capitalization of the sequences is inconsistent (if meaningful, what is the meaning?).

Agreed. We have removed the For/Rev designations for the primers.

In the ADM-2 deletions tab, what is the heading for column C? Column C primers are listed by name (from the first sheet) but the screening and sequencing primers are not all named. Does it make sense to include all primers in the "Primer list" tab and include their use in a different column?

We have reorganized S2 File and have strived to keep everything consistent. The first tab now lists all oligonucleotides by name used in the paper. The remaining tabs list each CRISPR-generated *adm-2* variant including the name and functional category of each oligo. The goal is to make it easy for someone else to analyze or replicate our methods, which we believe this accomplishes. New S3 File also provides the sequences (and surrounding sequence context) for every allele of *adm-2* in the paper.

Reviewer #2: The authors have been analyzing endocytic vesicle trafficking in molting. In their previous work, they found nekl-2 and nekl-3 protein kinases play important roles in this process. In this manuscript, Joseph et al. identified novel mutations in adm-2 as the suppressors for the molting and larval arrest defects of nekl-2 and nekl-3 double mutants (both are weak

alleles). Adm-2 encodes C. elegans ortholog of mammalian ADAM meltrin. ADM-2::GFP was broadly expressed and colocalized with endosomal and lysosomal markers in the hyp7 syncytium. The adm-2 suppressors failed to suppress the endocytic vesicle trafficking defects of nekl-2 and nekl-3 double mutants unlike their previously identified suppressor fcho-1. The authors found that the adm-2 affected the abundance of LRP-1 (sterol receptor) in the apical surface of hyp7; adm-2 loss-of-function increased LRP-1 levels, whereas adm-2 overexpression decreased them. Overall, this is an elegant genetic study analyzing the in vivo function of the ADAM family metalloprotease in animal development. Although all the experiments have been carefully conducted and the manuscript is well written, I think the presented data are still insufficient to support the authors' model that "loss of adm-2 suppresses molting defects in nekl mutants by eliminating a negative regulator of LRP-1, thereby compensating for defects in the efficiency of LRP-1 and sterol uptake".

We agree, and as described above, our model has changed substantially. Overall, we have scaled back on statements attributing LRP-1 de-repression as the major (or only) mechanism by which *adm-2* mutations suppress *nekl-2; nekl-3* molting defects. Our results, including new data, clearly indicate that the picture is more complex. Nevertheless, we think that the effect of ADM-2 on LRP-1 real, reproducible, and of interest. It also provides an example for how the removal of a negative regulator of molting could contribute to the suppression of molting defects and dovetails with previous work that we and others have carried out on the functions of LPR-1 and NEKLs in molting.

Comments:

1. Based on their observation that the amount of LRP-1::GFP is upregulated in the adm-2(fd300) and is downregulated in adm-2 overexpression, the authors proposed a model that ADM-2 negatively regulates LRP-1: The extracellular domain of LRP-1 is released by direct or indirect action of the ADM-2 protease. However, I feel that the observed effects are quite weak, and it is unclear whether LRP-1 is actually cleaved and shed from the membrane. Because GFP is fused to the Cterminus (cytoplasmic domain?) of LRP-1, it is conceivable that GFP can be still anchored to the plasma membrane even after ectodomain shedding. If the authors wish to present their model, they should examine the proteolytic cleavage of LRP-1, for example by Western blotting experiments and compare the amounts of cleaved fragments between these two experimental conditions. They also need to examine the colocalization of ADM-2 and LRP-1.

As described above, we have carried out these types of experiments, including the requested colocalization experiment. We found that ADM-2 and LRP-1 do partially co-localize in the worm skin (Fig 5J–M; Lines 314-315), consistent with the observed functional interaction. Our new data, however, also indicate that the effect of ADM-2 on LRP-1 is not attributable to ADM-2 protease activity and that additional functions/domains of ADM-2 likely play some role in the observed suppression by *adm-2* (Fig 6; S7 Fig). The picture to emerge is admittedly more complex, and we have adjusted our presentation, wording, and conclusions carefully

throughout to incorporate this view.

2. It is not convincing that the adm-2 mutation promotes sterol uptake because more membrane-bound LRP-1 is available in the mutant background. I think LRP-1 should be endocytosed and transported to the lysosomes for sterol to be released to the cytosol. The authors, however, observed that apical accumulation of LRP-1 in nekl-3::AID failed to be suppressed by adm-2(fd318) (even slightly enhanced).

I feel it might be possible that ADM-2 may act independently of LRP-1. Although the authors consider that ADM-2 inhibits molting through its negative regulation of steroid hormone biosynthesis, another possibility is that ADM-2 may inhibit the process downstream of the NHR hormone receptors or a hormone-independent process. I think this possibility should be addressed for example by examining whether the lrp-1 null mutation can fully block the suppressing effect of adm-2 on nekl-2; nekl-3 defects. Also, it will be interesting to see if adm-2 can suppress the molting phenotype of the lrp-1 null mutant. If the authors observe any improvement in molting in adm-2; lrp-1 double mutants, that means that adm-2 can act in an lrp-1-independent pathway.

We understand and concur with the reviewer's main issues, and as stated above, we have adjusted our statements accordingly throughout the manuscript. As suggested, we tested knockdown of LRP-1 in several contexts, although these studies did not provide any definitive mechanistic conclusions and so were not included in the revised manuscript. We found that (RNAi) knockdown of *adm-2* did NOT suppress molting defects in an *lrp-1* null mutant, which could be interpreted as consistent with ADM-2 acting through LRP-1. (We note that these RNAi conditions were identical to those in which we observed *nekl-2; nekl-3* suppression.) Likewise, we also tested knockdown of *lrp-1* by RNAi in wild-type, *adm-2* null mutants, and *nekl-2; nekl-3; adm-2* mutants. In all backgrounds the RNAi caused a fully penetrant molting defect, which could be argued as consistent with ADM-2 acting in part through LRP-1. However, this type of genetic argument is relatively weak and so we did not include this data.

3. The ADM-2::GFP is expressed in various tissues including hypodermis. Although the authors believe that loss of the adm-2 function in hypodermis is responsible for the suppression, it is still unclear. The authors need to experimentally determine the responsible tissues for example by expressing adm-2 using tissue-specific promoters in the adm-2; nekle-2; nekl-3 mutant background or tissue-specific adm-2 RNAi experiments in the nekl-2; nekl-3 mutant background.

We agree it's possible that ADM-2 could be affecting molting through its expression in non-hypodermal tissues, although this is not the most parsimonious explanation given our collective data. We have included wording to acknowledge this caveat in the text (Lines 214-217).

4. The patterns of expression of ADM-2 and LRP-1 were analyzed using mScarlet or GFP fusions. I think it is important to use functional fusion constructs to determine the protein localization. Please mention whether these constructs are functional or not.

As stated in the text, our expression studies were carried out using endogenously tagged CRISPR fusions of ADM-2 to GFP or mScarlet (e.g., Lines 193-195). We also did some analyses with an ADM-2::GFP fusion expressed from an extrachromosomal array (S3G Fig). This fusion was tested for rescue (de-suppression) of *nekl-2; nekl-3 adm-2* mutants and was shown to be functional (Fig 1F), indicating that the C-terminal fusion does not strongly alter ADM-2 function. Some additional wording has been added to clarify this point (Lines 152-154).

5. It is reported that the cytoplasmic domain of human ADAM13 is cleaved and translocated into the nucleus to regulate neural crest cell migration and that the cytoplasmic domain of ADM-2 can be functionally replaced with that of ADAM13. Because mutations either in the catalytic domain or the cytoplasmic domain of ADM-2 can act as suppressors, I am curious if the authors observed the localization of ADM-2::GFP or ::mScarlet in the nucleus.

We have not observed convincing nuclear localization of the ADM-2 reporters, although we looked quite hard for this. To further address this question we generated several new CRISPR variants in ADM-2 within a region just C-terminal to the TM domain. This region contains an overlapping (predicted) NLS and Furin cleavage site. Interestingly, we did observe considerable suppression with the NLS variant(s), potentially indicating a role for the nuclear localization of a cytoplasmic ADM-2 fragment (Fig 6, S9 Fig). However, we can't read too much into this because we don't know if this variant localizes correctly or if the domain-targeted CRISPR variants (in general) might be affecting the function of other domains of ADM-2. These caveats are discussed (Lines 370-377, 421-432).

6. Although the authors isolated various alleles in adm-2, detailed information for the mutation sites are provided for only part of the alleles. Please provide all the information if possible.

Detailed sequencing and context information is now provided for all the alleles of *adm-2* in the study. This is now referenced in the materials and methods (S3 File).

7. Fig. 4 M-O. Which focal plane do these photos depict?

Information regarding the focal plane (apical) is now provided in the figure (now Fig 3M–O).

8. Fig. S1. ADAM family members should be reworded as "ADAM and ADAMTS family members". Also, in the text.

Corrected.

Minor comments:

1. Fig. 3C, D. Please indicate a scale bar.

Now Fig 2C, D, Scale bar in Fig 2B is for Fig A–D. This is mentioned in the figure legend.

2. line 200. ADM-2 can be adm-2.

Changed to “loss of *adm-2* function” (Line 279).

3. line 226 S3D can be S3G.

Corrected (Line 214).

4. line 235-236. ADM-2::GFP is not shown in Fig 4A or 4C.

Corrected (Line 225).

5. line 265. ADM-2::mScarlet is not shown in Fig 5M-P.

Corrected (Line 250).

6. line 316.) can be missing.

This section was substantially altered (see above).

7. line 553. Heat shock methods for Fig 6F and G are not mentioned.

Corrected (Line 607).

Reviewer #3: Summary

This is a thoughtful and well-executed study of the mechanism of genetic suppression of nekl(rf) molting defects by the meltrin-family metalloprotease adm-2, revealing an interesting dynamic between negative and positive regulators of the molting process and clathrin-mediated endocytosis. The structure-function analysis of ADM-2 is impressively thorough. As described in greater detail below, the manuscript would benefit from:

1. inclusion of an fcho-1 control into panel 1G for comparison to adm-2 (and if possible, moving this panel to the start of figure 2).

We would prefer to refer readers to the previously published data in the Joseph et al., 2020 paper. We have now added a sentence to explicitly state the level of suppression observed for these alleles (Lines 263-264).

2. examination of adm-2-dependent LRP-1:GFP expression during molting (in place of, or to complement, the adult data in Figure 6f-m)

We addressed this question by assaying LRP-1::GFP levels in wild-type and *adm-2* mutant L4 larvae. Although not as robust as the differences seen in day-one adults, we did observe an increase in LRP-1::GFP in *adm-2* mutants (S7A Fig; Lines 298-299).

3. use of fluorescence-only images (rather than DIC overlay) in Fig 6b,c and inclusion of a nontransgenic control

The changes have been made.

4. Minor text edits for clarity, described below.

Details

1. Figure 2 Examines whether adm-2 suppression of nekl-2/3 double-rf larval molting defects could be due to restoration of CME. Panel G from figure 1 would work better at the start of figure 2, and benefit from the inclusion of an fcho-1 control for comparison (eg. fcho-1; nekl-2). This would provide clearer physiological support for the LRP-1 localization data of figure 2, which differs in both the stage (adult vs. larvae) and the genetic background (NEKL-3 AID vs. nekl-2/3(rf) from the phenotype (molting defect suppression). A note on the choice of this system for examination of LRP-1 expression (eg., rather than performing nekl-3AID from hatching, examining at the onset of molting defects) would also be helpful to the reader.

We thank the reviewer for the suggestion but feel that panel G from Fig 1 fits somewhat better with other panels showing genetic suppression. We also note that we specifically do the NEKL depletion studies in adults because depletion of NEKLs during larval stages causes molting defects, which can make the assessment of downstream effects on expression and localizatioin of markers quite difficult. For example, when we see defects in NEKL-depleted larvae it's hard to know whether these are directly due to NEKL depletion or might be secondary to defects in molting. Using adults gets around this issue. Note that the rationale for this approach was discussed quite extensively in a previous work (Joseph et al., 2020: 32069276).

2. Similarly, the examination of LRP-1:GFP expression in Figure 6 was performed in adults rather than larvae. I suggest adding an experiment examining adm-2-dependent changes in LRP-1 expression during molting, if possible. If LRP-1:GFP is below the limit of detection in larvae, it should be noted, with corresponding interpretations of adm-2 function expressed in more conservative language.

See directly above.

3. The heat shock dependence of the hs:ADM-4GFP transgene (Fig 6b,c) would be better illustrated by fluorescence-only images (black background) rather than fluorescence/DIC overlays, and by inclusion of a non-transgenic control.

See directly above.

4. Minor edits:

a. abstract: “Whereas loss of ADM-2 activity led to the upregulation of LRP-1, ADM-2 overexpression caused a reduction in LRP-1 abundance…” this sentence would benefit from an indication that this refers to the apical surface. eg) “Whereas loss of ADM-2 activity led to increased levels of apical LRP-1, ADM-2 overexpression caused a reduction in apical LRP-1 abundance…”

We have changed to abstract to “apical epidermal”.

b. lines 45-46: the phrasing makes it sound like adm-2 regulates something that in turn negatively regulates LRP-1. Re-phrase for clarity?

We note that this portion of the abstract was altered in response to our new findings.

c. line 101: remove the word ‘caused’ for clarity

Corrected (Line 106).

d. line 114: move the word ‘specifically’ to just before the word de-repressing

We note that this portion of the introduction was altered in response to our new findings.

e. For added transparency, the number of animals examined in 1E,F,G, 5c, 6a should be indicated.

We added this information to the requested PANELS. In addition, all raw data, including the numbers mentioned, are provided in full in S1 File. We now make sure to reference this in all the relevant figure legends.

f. line 126: change (Fig 1A and 1E) to just (Fig 1A), as there is no rescuing array in 1E and its description in line 128 is sufficient and appropriate.

Because of a low level of escapers (1-2%), it is possible to obtain GFP-minus adults in the original strain. Thus, we think it important to refer to 1E as this shows the quantification of the suppression effects.

g. line 136: change ‘The fd163 mutation’ to ‘The resulting intron retention’

Corrected (Line 142).

h. line 160: replace the semi-colons with commas

Corrected (Line 171-172).

i. line 178: replace the word ‘is’ with ‘may’ since this is based on lack of phenotypes from RNAi

Changed to “appears” (Line 189).

j. line 233-234: the words ‘rare’ and ‘occasionally’ are redundant with each other

Corrected (Line 222).

k. lines 237-239: the faintness of ADM-2 expression in hyp7 is postulated to be due to rapid turnover, possibly mediated by CME. This would make more sense to the reader if it were noted that detection at the plasma membrane was difficult to detect 'relative to other compartments'.

Corrected (Line 226).

l. line 320: the words ‘specifically’ and ‘only’ are redundant with each other

As discussed above, this portion of the manuscript was removed.

m. Figure 6 title could be more informative. Instead of “and LRP-1”, maybe “and apical abundance of LRP-1”?

Figure titles have been changed according to new data and emphases.

n. The font under the scale bars in panels 6C and E should be increased, or omitted altogether and described only in the figure legend, as is the case for panel D.

Corrected.

o. In Figure 6m, the Phsp-16::adm-2+ genotype is shortened to Padm-2+, but hs:adm-2+ or similar might be clearer.

Corrected.

Have all data underlying the figures and results presented in the manuscript been provided? Large-scale datasets should be made available via a public repository as described in the PLOS Genetics data availability policy, and numerical data that underlies graphs or summary statistics should be provided in spreadsheet form as supporting information.

Reviewer #1: Yes Reviewer #2: Yes Reviewer #3: Yes

PLOS authors have the option to publish the peer review history of their article (what does this mean?). If published, this will include your full peer review and any attached files.

If you choose "no", your identity will remain anonymous but your review may still be made public.

Do you want your identity to be public for this peer review? For information about this choice, including consent withdrawal, please see ourPrivacy Policy.

Reviewer #1: Yes: Tina L. Gumienny Reviewer #2: No Reviewer #3: No
